# A pain science education and walking program to increase physical activity in people with symptomatic knee osteoarthritis: a feasibility study

**DOI:** 10.1097/PR9.0000000000000830

**Published:** 2020-09-24

**Authors:** Tasha R. Stanton, Emma L. Karran, David S. Butler, Melissa J. Hull, Sarah N. Schwetlik, Felicity A. Braithwaite, Hannah G. Jones, G. Lorimer Moseley, Catherine L. Hill, Christy Tomkins-Lane, Carol Maher, Kim Bennell

**Affiliations:** a IIMPACT in Health, Allied Health and Human Performance, The University of South Australia, Adelaide, South Australia, Australia; b Neuroscience Research Australia, Sydney, New South Wales, Australia; c Neuro Orthopaedic Institute Australasia PTY LTD, Adelaide, South Australia, Australia; d Alliance for Research in Exercise, Nutrition and Activity (ARENA), Allied Health and Human Performance, The University of South Australia, Adelaide, South Australia, Australia; e Department of Psychology, University of Bath, Bath, United Kingdom; f Rheumatology Department, The Queen Elizabeth Hospital, Adelaide, South Australia, Australia; g Faculty of Health and Medical Sciences, Adelaide Medical School, The University of Adelaide, South Australia, Australia; h Department of Health and Physical Education, Mount Royal University, Calgary, AB, Canada; i Centre for Health, Exercise and Sports Medicine, Department of Physiotherapy, The University of Melbourne, Victoria, Australia

**Keywords:** Osteoarthritis, Pain, Physical activity, Walking program, Pain science education, Sham ultrasound

## Abstract

**Introduction:**

Nine of 10 people with knee osteoarthritis are inactive. Unhelpful pain beliefs may negatively influence physical activity levels. Targeting these unhelpful pain beliefs, through contemporary pain science education (PSE), may provide benefit.

**Objectives:**

To evaluate the feasibility of conducting a clinical trial to determine the effect of adding PSE (vs adding sham ultrasound) to an individualised, physiotherapist-led education and walking program in people with painful knee osteoarthritis.

**Methods:**

Twenty participants were randomised (1:1) into the PSE group or Control group, each receiving 4 in-person weekly treatments, then 4 weeks of at-home activities (weekly telephone check-in). Clinical outcomes and physical activity (7 days of wrist-worn accelerometry) were assessed at baseline, 4 (clinical outcomes only), 8, and 26 weeks. *A priori* feasibility criteria for recruitment, intervention adherence, viability of wrist-based accelerometry, and follow-up retention were set. Perceived intervention credibility, acceptability, and usefulness from participants and clinicians were assessed (ratings, written/verbal feedback).

**Results:**

Most feasibility criteria were met. On average, 7 adults/wk were eligible, with 70% recruited. Treatment compliance was high (in-person: 80% PSE; 100% Control; at-home: 78% PSE; 75% Control). Wrist-based accelerometry had >75% valid wear-time. Sufficient follow-up rates were not achieved (26 weeks: 65%). Participant and clinician feedback highlighted that PSE was too complex and did not match patient expectations of “physiotherapy”, that sham ultrasound was problematic (clinician), but that both treatments had high credibility, acceptability, and usefulness.

**Conclusions:**

Progression to a full trial is warranted. Strategies to increase participant retention, refine the PSE content/delivery, and replace/remove the sham intervention are required.

## Abstract 2

Supplemental Digital Content is Available in the Text.

This feasibility study of contemporary pain science education to increase activity levels in people with painful knee osteoarthritis supports progression to a larger trial.

## 1. Introduction

Osteoarthritis (OA) is a leading cause of pain and disability worldwide.^10^ Symptomatic knee OA is especially disabling, resulting in reduced independence and quality of life.^2^ Regular structured physical activity reduces pain and disability in people with symptomatic knee OA,^6,15^ including those with end-stage OA awaiting joint replacement.^42^ Even small increases in physical activity predict improved function^12^ and reduced disability.^36^ Nonetheless, 9 in 10 people with painful knee OA are inactive.^43^

People with OA who are inactive are more likely, than those who are active, to believe that they are unable to exercise and that activity is unsafe.^11,44^ People with knee OA also focus heavily on pain and believe OA is an incurable, progressive, “bone-on-bone” disease^32^ caused by “wear-and-tear”.^7^ This is despite strong evidence that physical activity does not further damage the joint.^6,34^ Such beliefs (eg, “pain during activity represents more joint damage”) negatively influence patients' acceptance of undertaking evidence-based treatment such as exercise,^7^ and reduce their participation in (potentially) pain-provoking activity.^20^

Most current approaches to increasing physical activity encourage “movement despite pain” (eg, behavioural interventions^45^ that use coping skills, with the “promise” of pain-relieving effects over time). However, such approaches may seem counterintuitive to patients as long as they consider pain to be a marker of damage.^28^ Contemporary pain science education (PSE) was developed to shift the meaning of pain from that of a marker of tissue damage (more pain means more damage) to that of a need to protect the body from real or perceived danger.^28^ In this way, PSE provides a scientific basis for a biopsychosocial model of pain and disability and the enhanced sensitivity generated by central nervous system adaptations as pain persists.^28^

Meta-analyses and randomised controlled trials (RCTs) show that PSE increases pain knowledge, reduces unhelpful pain beliefs,^26,29^ and improves pain, function, and disability across several musculoskeletal pain states,^25,26,29,39,46^ but knee OA-specific data are lacking. Our clinical audit data from people with OA-associated knee pain (n = 139) showed that 4 weeks of physiotherapist-led PSE, followed by individualised functional/activity goals, significantly improved self-rated activity (*P* < 0.001), pain, and catastrophising (both *P* < 0.01) at 6 and 12 months after treatment (unpublished). Experimental evidence to confirm these findings is now needed, particularly using more robust, objective measures of physical activity (given limited validity of self-report^33^) and using a control group with a credible sham intervention component to offset the extra education time PSE requires.

Before embarking on a large-scale trial, feasibility should be confirmed. Therefore, this study aimed to determine the feasibility (participant eligibility/recruitment, intervention adherence, objective physical activity assessment compliance, and retention to long-term follow-up) of an RCT investigating the effect of adding PSE (vs adding sham ultrasound) to an individualised, physiotherapist-led general education and walking program for people with painful knee OA. Acceptability of PSE content and its delivery format (for participants and clinicians) was examined, as was the Control intervention credibility. Secondary objectives were to identify barriers to participation and to provide within-group treatment effect estimates.

## 2. Methods

### 2.1. Study design/setting

A randomised, parallel group, assessor-blinded, sham-controlled feasibility trial was undertaken at the University of South Australia (UniSA) Clinical Trials Centre, Adelaide, from July 2018 to February 2019. This study was approved by UniSA's Human Research Ethics Board (ID200791) and prospectively registered with the Australian and New Zealand Clinical Trials Registry (ACTRN12618001149257). Participants were randomly allocated (1:1) to groups through randomisation schedule (Excel) with random permutated blocks of 4 and 6. Allocation was concealed in sequentially numbered, sealed, opaque envelopes created by an investigator not involved in the study. Participants were allocated to the groups by an independent investigator who coordinated treatment scheduling.

Participants were advised that they would receive 1 of 2 physiotherapy treatments aiming to improve overall health (limited disclosure). Study questionnaires (baseline, 4 weeks) were administered in-person by an independent researcher, blinded to group assignment. Treating clinicians were unavoidably aware of group assignment, but were not involved in outcome assessment.

### 2.2. Participants

People aged 50 years and older with painful knee OA^1^ were recruited from the community in South Australia through local newspapers, Arthritis Australia newsletters, and social media (July–August 2018). We aimed to recruit 20 participants (n = 10/group). See Table 1 for eligibility criteria.

**Table 1:** Eligibility criteria.

Inclusion	Exclusion
Painful knee OA (diagnosed by a medical professional and met ACR clinical criteria) >6 mo OA duration ≥40/100 average knee pain over the past wk: overall and/or during walking (0–100 NRS)	Conditions preventing safe participation in physical activity Neurological disorders affecting the lower limb Inflammatory arthritis Fibromyalgia Cognitive impairment Severe depression (>21 on DASS) Recent intra-articular therapy (past 3 mo) Previous knee replacement (painful knee) or planned knee replacement or surgery (next 6 mo) Moderate/vigorous activity levels above guideline recommendation (>150 min/wk; IPAQ-SF) No radiograph or other imaging report of their affected knee (and unwilling to get one)

ACR, American college of rheumatology; DASS, depression, anxiety and stress scale; IPAQ-SF, International Physical Activity Questionnaire—Short-Form; mo, month; OA, osteoarthritis; NRS, numeric rating scale.

### 2.3. Interventions

The interventions have been described in accordance with the TIDieR Checklist.^17^

#### 2.3.1. General intervention content

**Figure 1.**
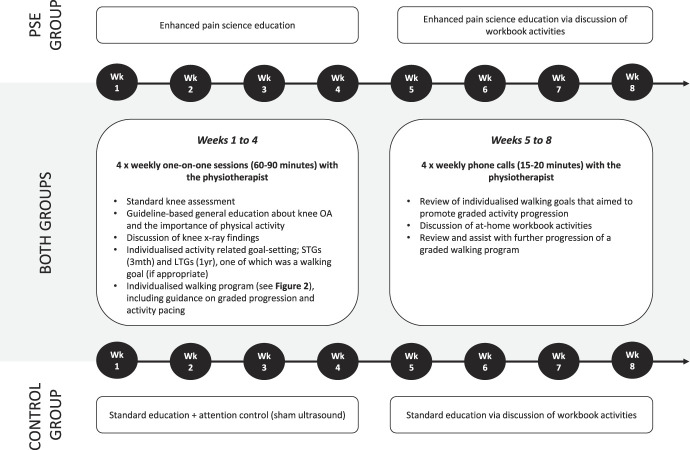
Details of study interventions. In both groups, the in-person and at-home treatment durations were inclusive of both the general content (provided in both groups) and the group-specific content. LTGs, long-term goals; OA, osteoarthritis; PSE, pain science education; STGs, short-term goals.

**Figure 2.**
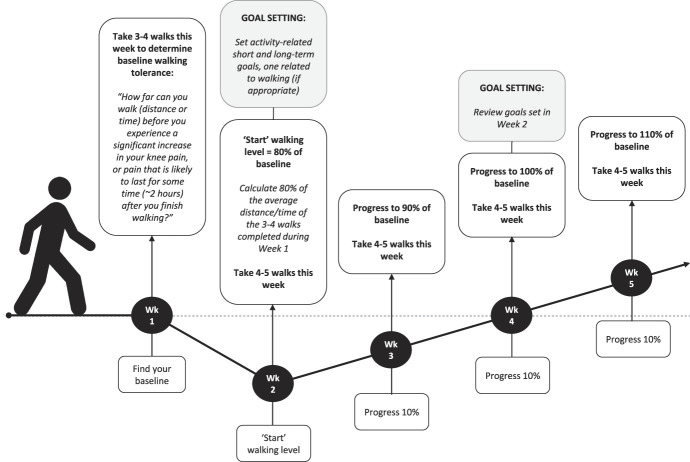
Graded walking program and goal setting procedure undertaken in both intervention groups. The number of walks per week was individualised to the participant, but in all cases, during week 2, we aimed to add one additional walk per week at a lower time/distance (ie, 4–5 walks per week is provided as an example here). At 8 weeks (not shown here), the walking program was reviewed and progressed (or maintained) for the following 4 weeks. Short-term and long-term activity goals were also reviewed at 8 weeks.

Participants in both groups were provided with guideline-based general OA and physical activity education^23^ in addition to an individualised walking program. A summary of the in-person and at-home treatment sessions is provided in Figure 1 and that of the walking program in Figure 2.

#### 2.3.2. Group-specific content

Differences between intervention groups related to the educational content provided (Table 2) and the use of sham ultrasound in the Control group.

**Table 2:** Education features of the intervention groups.

	Enhanced education—PSE	Standard education—Control
Overall objective(s)	To shift participants' conceptualisation of pain from that of a marker of tissue damage to that of a marker of the perceived need to protect the body. To educate that pain is a protective feature of our system, not a “damage-meter”; thus, pain can be modulated by other things besides tissue damage and danger messages (ie, nociception).	To increase participants' knowledge about OA and the importance of physical activity in reducing osteoarthritic pain and increasing general health.
Pain education topics	Basic nervous system anatomy/function; distinction between nociception and pain; protective function of pain; peripheral/central sensitization; upregulation of brain mechanisms that serve protection; the state of “hyperprotection” offered by normal biological adaptations; the concept of an internal “Protectometer” (modulated by multifaceted danger and safety cues).	Basic OA and pain information as per the Arthritis Australia handbook.
Activity education	That physical activity does not increase joint damage but does have wide-ranging health benefits and OA-specific benefits. That physical activity is key to bioplasticity—ie, inducing changes in our system—and that it decreases overprotectiveness of our system, a change that often occurs with persistent pain.	That physical activity has wide-ranging health benefits as well as OA-specific benefits and that even people with severe OA benefit.
X-ray interpretation	The aim is to ‘dethreaten’ radiological findings. A detailed analysis of participants' own x-ray was undertaken, focusing on positive features (eg, excellent bone density) using standardised wording. Education about the poor correlation between x-ray findings and pain was provided.	The aim was to discuss radiological findings, focusing on the interpretation section as would occur in regular practice. Focus was on discussing the x-ray features that resulted in participants receiving a diagnosis of OA.

OA, osteoarthritis; PSE, pain science education.

##### 2.3.2.1. Pain science education group

###### 2.3.2.1.1. Weeks 1 to 4, one-on-one sessions

Participants received PSE, which expanded upon routinely provided information about OA and activity (See Supplementary File 1, available at http://links.lww.com/PR9/A67). Pain science education was based on contemporary pain science understanding, aiming to reduce the conviction that knee pain was an accurate marker of the knee's vulnerability to damage by incorporating belief revision strategies of conceptual change science.^28^ These strategies included challenging existing knowledge and refining learning strategies for new concepts through applying principles of multimedia learning.^28^ Participants received the “Explain Pain”^8^ and the “Protectometer”^27^ books (Noigroup Ltd, Adelaide, Australia), which both discuss pain concepts from this intervention. Participants were given at-home reading from the books and relevant multimedia content, which was revisited at the subsequent session to explore understanding (Supplementary File 1, available at http://links.lww.com/PR9/A67).

###### 2.3.2.1.2. Weeks 5 to 8, at-home treatment session

Participants' weekly tasks included using the Protectometer^27^ to identify the unique safety and danger cues for activity that could influence pain, brainstorming active vs passive coping strategies, exploring how to target individual features that could influence pain (conceptualised as “Danger in Me”, “Safety in Me” brain networks), and a pain knowledge quiz.

##### 2.3.2.2. Control group—standard education and sham ultrasound

###### 2.3.2.2.1. Weeks 1 to 4, one-on-one sessions

Participants received 4 sessions of “standard” information about knee OA and activity (using the Arthritis Australia handbook/resources; Table 2). To match time with the treating therapist between groups, this group also received sham treatment in the form of inactive ultrasound (as per previous work^5^), during which the clinician engaged the participant in general conversation. If participants discussed their knee pain and/or related concerns, the clinician was instructed to only offer advice and/or information consistent with the Arthritis Australia resource for knee OA. Supplementary File 2 provides the session-specific intervention breakdown used to match therapist-time between groups (available at http://links.lww.com/PR9/A67).

###### 2.3.2.2.2. Weeks 5 to 8, at-home treatment sessions

Participants received a workbook with weekly activities that included information and questions about the known benefits of activity, health risks of inactivity, and the relevance to OA.

### 2.8. Treating physiotherapists

Two physiotherapists delivered the study interventions, each providing only one of the interventions to reduce therapeutic cross-over between groups. The clinician providing PSE had ∼10 years of clinical experience, attended the Noigroup Explain Pain course (www.noigroup.com/), and received ∼20 hours of in-depth training from PSE Expert (D.S.B.). The clinician providing the Control treatment had ∼3 years of clinical experience and received ∼8 hours of training (T.R.S.) on educational content and sham ultrasound provision. Both therapists received 1 hour of training for the walking program (T.R.S., E.L.K.).

### 2.9. Procedure and data collection

**Figure 3.**
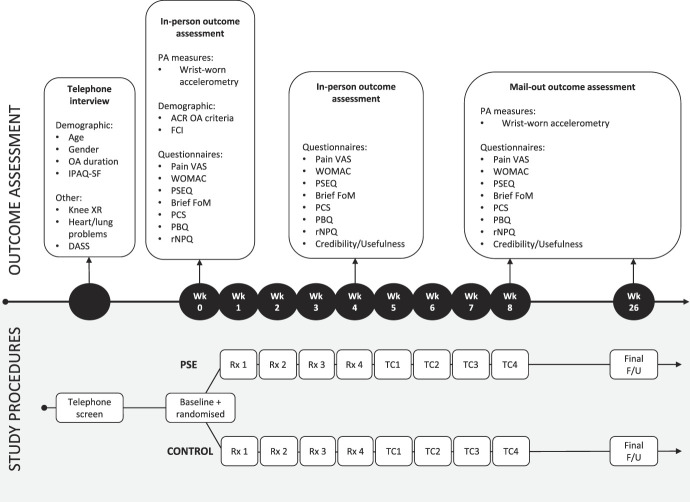
Study procedure, treatment sessions, and outcome assessment timing. ACR OA criteria, American College of Rheumatology Osteoarthritis clinical diagnosis criteria; DASS, depression, anxiety, and stress scale; F/U, follow-up; IPAQ-SF, International Physical Activity Questionnaire—Short Form; Knee XR, knee x-ray or other imaging report; PA Measures, physical activity measures; PSE, pain science education; Pain NRS, average pain intensity over the last week (at rest and while walking) using a 0 to 100 numerical rating scale (only used for initial telephone screening); Pain VAS, average pain intensity over the last week (at rest and while walking) via 0–100 mm visual analogue scale; PBQ, pain beliefs questionnaire^13^; PSEQ, pain self-efficacy questionnaire^30^; Brief FoM, brief fear of movement scale for OA^35^; PCS, pain catastrophizing scale^38^; PSFS, patient-specific functional scale^37^; rNPQ, revised Neurophysiology of Pain Questionnaire measuring pain knowledge^9^; Rx, treatment; TC, telephone call; WOMAC, the Western Ontario McMaster Universities OA Index.^3^

Volunteers underwent an initial telephone screen for basic eligibility criteria (diagnosis of knee OA by a medical practitioner, pain NRS ≥40/100, and absence of heart/lung conditions) by an administrative officer at the Clinical Trials Centre, followed by in-depth telephone screening by study researchers (T.R.S., E.L.K.) (Fig. 3). Eligible participants were scheduled for an appointment at the UniSA Clinical Trials Centre, where written informed consent was obtained.

Participants completed a baseline questionnaire that included demographic information (age, sex, height, and weight), comorbidities (Functional Comorbidity Index^16^), and clinical outcome measures^3,9,13,30,35,37,38^ (Fig. 3) based on OARSI recommendations for clinical trials of knee OA.^24^ Objective physical activity levels were assessed through wrist-worn accelerometry (GT9X, Actigraph LLC, Pensacola, FL; initialised at 50 Hz, 60-second epochs, duration: 14 days). After baseline assessment, participants were provided with the accelerometer watch and instructed to wear it 24 hr/d for 7 days on the nondominant wrist, recording any time that they removed it in a logsheet. Participants returned the accelerometer and logsheet at their first intervention visit (1 week after baseline assessment). The PSE group in-person sessions were audio-recorded for future in-depth analysis to guide intervention modifications.

Follow-up assessments occurred at 4, 8, and 26 weeks. The 4-week assessment was completed in-person (same blinded assessor as at baseline), and occurred directly after each participant's final in-person intervention. The 8- and 26-week assessments were undertaken through reply paid mail-out (paper questionnaires and accelerometer/logsheet).

### 2.10. Primary outcomes

#### 2.10.1. Feasibility outcomes

*A priori* decision-making criteria were used to determine feasibility and the ability to progress to a full RCT for outcomes of recruitment/eligibility rate, intervention adherence, compliance with objective measures of physical activity, and retention at follow-up assessments (Table 3). Participant recruitment rates were calculated by recording the number of participants per week (on average) identified as eligible and the number of eligible participants who agreed to participate. Intervention adherence was operationalised as the proportion of participants completing in-person and at-home treatments, and the proportion receiving full treatment content (covered all topic areas), calculated from attendance logs and the physiotherapist's notes. Compliance with the accelerometry protocol was calculated at each time point as the proportion of participants with valid accelerometry wear-time (defined as at least 4 days of ≥10 hours of waking hour wear-time). Retention to follow-up was calculated as those with valid questionnaire data for each time point.

**Table 3:** Progression criteria decision aid to lead to a full clinical trial.

	Decision criteria to proceed to full clinical trial		
	Proceed	Proceed with protocol amendments	Do not proceed
1. Recruitment and eligibility	At least 1 adult (on average) per week can be identified as eligible for inclusion	Less than 1 adult per week (on average) can be identified as eligible for inclusion.	Less than 1 adult per fortnight (on average) can be identified as eligible for inclusion.
	1 (or more) in 4 eligible participants recruited	At least 1 in 6 eligible participants recruited	Less than 1 in 6 eligible participants recruited
2. Intervention adherence	75% or more of participants randomised to PSE attend at least 3 intervention sessions	At least 50% of participants complete at least 3 intervention sessions	Less than 50% of participants complete 3 intervention sessions
	60% or more of at-home treatments are completed	At least 50% of at-home treatments are completed	Less than 50% of at-home treatments are completed.
	At least 80% of interventions provided in full (all content covered)	At least 50% of interventions provided in full (all content covered)	Less than 50% of interventions provided in full (all content covered)
3. Compliance with objective physical activity assessment	70% or more of participants have at least 4 d of ≥10 hr of valid wear time (accelerometry)	At least 50% of participants have at least 4 d of ≥10 hr of valid wear time (accelerometry)	Less than 50% of participants have at least 4 d of ≥10 hr of valid wear time (accelerometry)
4. Retention at long-term follow-up assessments	75% or higher follow-up rate at 26 wk	At least 50% follow-up rate at 26 wk	Less than 50% follow-up rate at 26 wk

PSE, pain science education.

#### 2.10.2. Intervention acceptability

Participants' and treating clinicians' perspectives on the acceptability of the clinical interventions were gathered. Intervention format, content acceptability and usefulness, as well as perceived credibility were assessed using a purpose-designed Participant Experience Questionnaire (PEQ; 5-point Likert scale ranging from “strongly agree” to “strongly disagree”), short-answer questions at 4, 8, and 26 weeks, and audio-recorded telephone interviews at 4 and 8 weeks (Supplementary File 3, available at http://links.lww.com/PR9/A67). Control participant's PEQ credibility ratings were used to assess sham ultrasound credibility. Short-answer questions and interviews explored what participants liked the most/least about the treatment, and their suggestions for the content and format of the sessions.

Treating clinicians judged the perceived acceptability of the intervention to the participant at weeks 4 and 8 (Do you think the participant found this to be an acceptable intervention? Yes/No), and, at trial conclusion, completed 4 short-answer questions, supplemented by verbal interview, about their experience delivering the treatment and on content and format (Supplementary File 3, available at http://links.lww.com/PR9/A67).

### 2.11. Secondary outcomes

These aimed to identify barriers to participation (reasons for eligible participants declining study involvement) and to report within-group change scores for the clinical and physical activity outcome measures.

### 2.12. Data handling and statistical analysis

Feasibility outcomes were examined in terms of frequencies and percentages. Intervention acceptability was determined based on the proportion of participants in each group who rated “agree” or “strongly agree” for treatment acceptability, usefulness, and credibility (average of 3, 4, and 3 PEQ questions, respectively). Participant feedback from the short-answer questionnaires and the audio-recorded phone calls regarding treatment were transcribed (audio) and summarised through content analysis^4^ by an independent experienced qualitative researcher (M.J.H.). Frequency of descriptive themes occurring within each group for each response question was counted, using multicoding for longer responses.^31^ Manifest analysis was chosen to avoid adding perceptions beyond those intended by the participants (researcher performing the analysis did not communicate with participants).^4^

As per our study protocol, only *within-group* change scores (and 95% confidence intervals) were calculated for clinical and activity outcomes from baseline to each follow-up point using available data (sensitivity analyses used imputation with the baseline value carried forward). Two physical activity analyses were performed (Actilife software; “worn-on-wrist” scaling method for Troiano cut-points^22,41^): (1) average daily step count and (2) average daily minutes at sedentary/light/moderate/vigorous activity levels.^41^

## 3. Results

**Figure 4.**
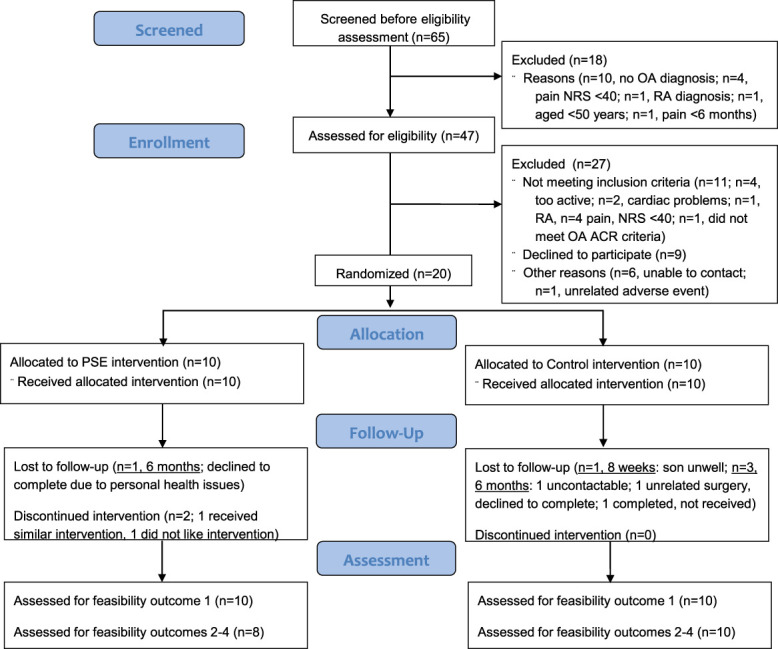
The CONSORT flow diagram for feasibility studies. ACR, American College of Rheumatology; NRS, numeric rating scale; OA, osteoarthritis; PSE, pain science education; RA, rheumatoid arthritis.

A total of 141 people expressed interest in the study. Of the 65 that underwent telephone screening, 47 underwent full eligibility screening. Of these 47, 11 were ineligible, 9 declined to participate and 6 were unable to be contacted, leaving 21 eligible for inclusion (Fig. 4). One participant had an unrelated adverse event (angina requiring hospitalisation) after baseline assessment but before randomisation, and was excluded (resulting in 10/group). Two PSE group participants withdrew within the first 2 sessions: one reported having received a similar intervention for his back and therefore saw minimal additional value in the program, and one reported that education did not align with her expectations of physiotherapy treatment. Table 4 provides baseline participant demographics and clinical measures.

**Table 4:** Participant demographics and baseline outcomes.

	PSE (n=10)	Control (n=10)	Overall (n=20)
Age	69.2 (6.5)	64.8 (7.9)	67.0 (7.4)
Gender (count)	6 female	8 female	14 female
Height	168.2 (10.7)	167.3 (9.6)	167.8 (9.9)
Weight	86.0 (20.5)	87.4 (18.5)	86.7 (19.0)
BMI	30.5 (7.4)	30.6 (6.8)	30.5 (6.9)
Education (count)			
Did not complete high school	2	0	2
High school	2	4	6
Nonuniversity qualification	5	2	7
University qualification	1	1	2
Postgraduate degree	0	1	1
Bilateral knee pain (count)	8	5	13
Most painful knee (count)			
Left	4	3	7
Right	3	7	10
Same	2	0	2
Varies	1	0	1
Duration of pain for most painful knee (count)			
6–12 mo	4	3	7
1–2 y	1	2	3
2–5 y	1	2	3
5–10 y	1	1	2
10–20 y	3	0	3
>20 y	0	2	2
Duration of pain for least painful knee (count)			
6–12 mo	1	1	2
1–2 y	3	0	3
2–5 y	0	2	2
5–10 y	1	1	2
10–20 y	3	0	3
Duration of activity limitation due to knee (count)			
<6 mo	1	1	2
6–12 mo	3	1	4
1–2 y	2	3	5
2–5 y	2	2	4
5–10 y	1	1	2
10–20 y	1	0	1
>20 y	0	1	1
Missing	0	1	1
Other knee symptoms (count)	8	10	18
Stiffness (count)	8	8	16
Bothersomeness (VAS)	4.8 (1.4)	5.3 (2.3)	5.0 (1.9)
Clicking (count)	7	7	14
Bothersomeness (VAS)	4.3 (2.7)	6.1 (1.7)	5.2 (2.4)
Pins and needles, tingling (count)	2	0	2
Bothersomeness (VAS)	5.5 (0.81)	—	5.5 (0.81)
Weakness (count)	7	5	12
Bothersomeness (VAS)	6.0 (1.0)	6.7 (2.2)	6.3 (1.6)
Giving way (count)	6	5	11
Bothersomeness (VAS)	5.0 (3.1)	5.5 (2.4)	5.2 (2.6)
Avg pain most painful knee (VAS)	5.9 (1.9)	6.0 (2.3)	6.0 (2.0)
Avg pain walking most painful knee (VAS)	6.2 (1.5)	6.5 (1.6)	6.3 (1.5)
Avg pain least painful knee (VAS)	4.1 (2.2)	4.9 (2.3)	4.4 (2.2)
Avg pain walking least painful knee (VAS)	4.4 (2.7)	4.5 (4.1)	4.4 (3.1)
FCI	3.6 (2.4)	2.7 (1.5)	3.2 (2.0)
WOMAC			
Pain subscale	12.2 (4.0)	10.8 (2.4)	11.5 (3.3)
Function subscale	42.8 (9.8)	30.6 (12.0)	36.7 (12.4)
Total	55 (13.4)	41.4 (13.9)	48.2 (15.0)
PSFS			
Activity 1	3.2 (2.2)	4.2 (1.1)	3.7 (1.8)
Activity 2	3.0 (1.7)	3.3 (2.5)	3.2 (2.1)
Activity 3	2.9 (1.9)	3.8 (1.6)	3.3 (2.2)
PSEQ	37.4 (10.1)	47.0 (8.6)	42.2 (10.4)
Brief FoM	8.4 (4.3)	8.0 (4.8)	8.2 (4.4)
PCS	12.3 (14.5)	11.3 (12.1)	11.8 (13.0)
PBQ			
Organic	14.3 (5.8)	18.3 (4.1)	16.3 (5.3)
Psychological	9.0 (5.2)	9.6 (3.9)	9.3 (4.5)
rNPQ	3.4 (2.0)	4.1 (2.1)	3.8 (2.1)
DASS			
Depression	6.6 (7.8)	7.0 (9.4)	6.8 (8.4)
Anxiety	9.8 (11.9)	4.2 (5.7)	7.0 (9.5)
Stress	9.2 (9.8)	11.6 (9.8)	10.4 (9.6)
Average daily step count (steps/d)	10364 (1745)	11232 (2844)	10898 (3369)
Average daily activity count (minutes/d)			
Sedentary	742 (69)	734 (103)	738 (88)
Light	511 (74)	577 (75)	545 (75)
Moderate	104 (38)	107 (59)	106 (50)

Values are mean and SD unless otherwise indicated.

BMI, body mass index (<18.5 underweight; 18.5–24.9 normal; 25.0–29.9 overweight; ≥30 obese); Brief FoM, brief fear of movement scale (6 items, 0–3 likert scale, maximum score 18, lower score represents less fear); DASS, depression anxiety and stress scale (depression subscale: 7 items, 0–3 Likert scale, total score is doubled, maximum score 42, lower score represents fewer depressive symptoms; anxiety subscale: 7 items, 0–3 Likert scale, total score is doubled, maximum score 42, lower score represents fewer anxiety symptoms; stress subscale: 7 items, 0–3 Likert scale, total score is doubled, maximum score 42, lower score represents fewer stress symptoms); FCI, Functional Comorbidity Index (18 items, maximum score 18, lower score represents fewer comorbid conditions); PCS, Pain Catastrophizing Scale (13 items, 0–4 Likert scale, lower score represents less catastrophizing); PBQ, Pain Beliefs Questionnaire (organic subscale: 8 items, 0–5 Likert scale, lower score represents more unhelpful pain beliefs; psychological subscale, 4 items, 0–5 Likert scale, lower score represents more unhelpful pain beliefs); PSE, pain science education; PSFS, patient-specific functional scale (participants chose up to 5 activities and rated their ability to perform each activity on a scale of 0–10, lower score represents less ability to perform activity; top 3 activities reported here); PSEQ, Pain Self-Efficacy Questionnaire (10 items, 0–6 Likert scale, maximum score 60, lower score represents lower confidence); rNPQ, revised neurophysiology of pain questionnaire (13 items, maximum score 13, lower score represents fewer correct responses); VAS, visual analogue scale for bothersomeness (0–100 mm, lower score represents less bothersomeness) and for pain (0–100 mm, lower scores represent less pain); WOMAC, Western Ontario McMaster Universities OA Index (pain subscale: 5 items, 0–4 Likert scale, maximum score 20, lower score represents lower pain levels; physical function subscale: 17 items, 0–4 Likert scale, maximum score 68, lower score represents less difficulty; total WOMAC score is the sum of the 2 subscales).

### 3.1. Feasibility outcomes

#### 3.1.1. Recruitment and eligibility

Both feasibility criteria were met. Given the treating clinicians' availability, the sample was recruited in 2 blocks of 10. In block one, 18 adults were identified as eligible within 2 weeks (mean: 9/week) and in block 2, 10 adults (mean: 5/week) were eligible, giving an overall average of 7 adults/week (Table 3). Of those eligible, 7 in 10 were recruited (70%), which translates to 2.8 out of 4 participants.

#### 3.1.2. Intervention adherence: in-person, at-home, and overall content

These 3 feasibility criteria were met in both groups.

##### 3.1.2.1. In-person and content

In the PSE group, 80% of participants attended at least 3 in-person sessions. When excluding the 2 participants who withdrew, 100% attended at least 3 intervention sessions. Content was covered in full in 97% of sessions.

In the Control group, all participants attended all 4 sessions. Treatment content was fully completed (all education sections of Arthritis Australia handbook and 20 minutes of sham ultrasound/session).

##### 3.1.2.2. At-home

In the PSE group, 97% of weekly telephone calls were made (31 of 32 total weekly calls to 8 participants; 78% of total sample) and 78% of self-guided workbook activities were completed (25 of 32; 63% of total sample). The weekly walking goal was achieved in 59% of participants (19 of 32 interactions; 48% of total sample) and was attempted in 39% (12 of 32; unsure for n = 1, unable to contact at week 8). Walking goals were not met by one participant during week 2 due to an unrelated adverse event (fall from chair with hip bruising).

In the Control group, 95% of weekly telephone calls were made (38 of 40 total weekly calls to 10 participants) and 75% of self-guided workbook activities were completed (30 out of 40 total workbook activities; n = 2 unclear; n = 3 not recorded; n = 5 forget/too busy). The weekly walking goal was achieved in 73% of participants (29 of 40 interactions) and was attempted in 15% (6 of 40; unsure for n = 2, as unable to contact). The walking goal was not attempted in 8% (3 of 40; n = 1 on holiday; n = 2 too busy).

#### 3.1.3. Compliance with objective physical activity assessment

This feasibility criterion was met: >75% of participants (at all time points) had valid accelerometer wear-time. At baseline, all participants had valid wear-time. At 8 weeks, 89% (16 of 18 participants; 80% given total sample) had valid wear-time; activity data were missing in one participant from each group (device malfunction in PSE participant: no data file). At 26 weeks, of retained participants (n = 14; Control: 2 lost to follow-up; PSE: 2 lost to follow-up, 2 withdrew), 100% had valid wear-time. Valid wear-time at 8 and 26 weeks considering the full sample (n = 20) was 80% and 70%, respectively.

#### 3.1.4. Retention at long-term follow-up assessment

This feasibility criteria of >75% follow-up at 26 weeks was not met. At 4 weeks, both groups had 100% follow-up (questionnaire completion in 18 of 18). At 8 weeks, follow-up was 100% of in the PSE group (8 of 8; 80% given total sample) and 90% in the Control group (9 of 10). At 26 weeks, follow-up was 88% in the PSE group (7 of 8; 70% given total sample) and 60% in the Control group (6 of 10), resulting in an average retention rate of 72% across groups. Considering the whole sample (n = 20), this is a retention rate of 65% at 26 weeks.

### 3.2. Acceptability, usefulness, and credibility of interventions

#### 3.2.1. Participant ratings and feedback

**Figure 5.**
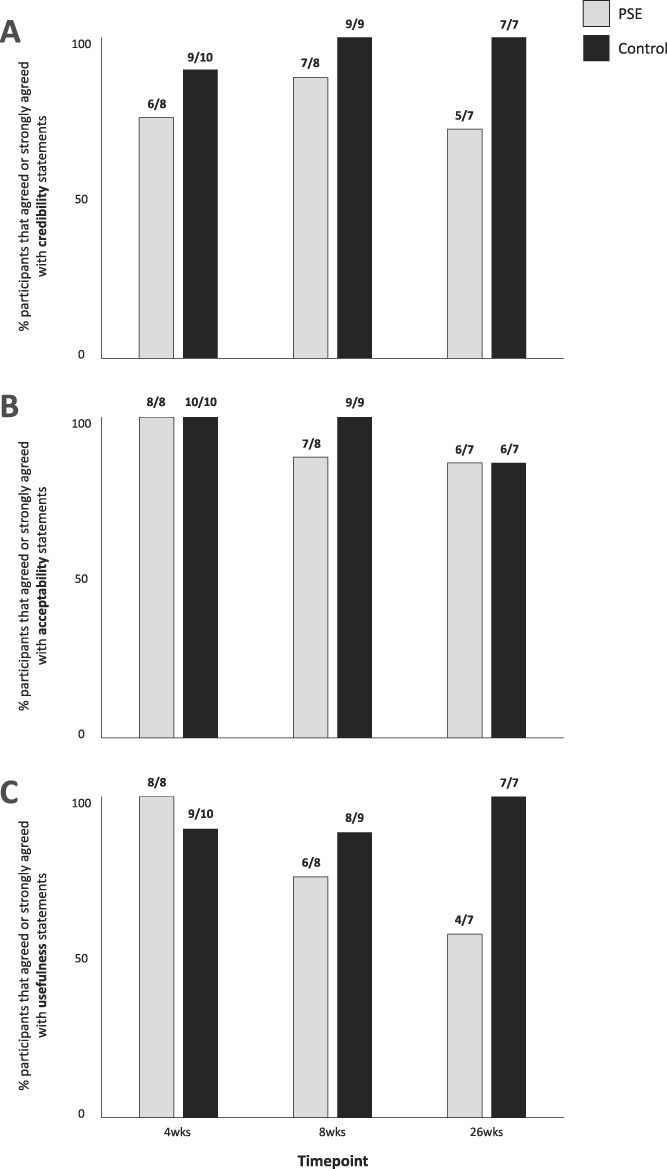
Participant ratings of the study interventions. (A) Perceived credibility; (B) perceived acceptability; (C) perceived usefulness. PSE, pain science education.

At 4 and 8 weeks, ≥75% of participants in both groups either agreed or strongly agreed with treatment credibility, acceptability, and perceived usefulness statements, suggesting strong support of the treatments (Fig. 5). At 26 weeks, these ratings were lower in the PSE group (71%, 86%, and 57%, respectively) than the Control group (100%, 86%, 100%, respectively). Participant feedback through short answer/interview identified that 2 participants in the PSE group did not consider it to be “treatment.”

Perceived intervention credibility in Control group participants was high at all time points (Fig. 5A): >89% of participants agreed/strongly agreed with the statements, which speaks to the credibility of sham ultrasound. Short-answer responses (Supplementary File 4, available at http://links.lww.com/PR9/A67) and verbal interviews (Supplementary File 5, available at http://links.lww.com/PR9/A67) showed that participants liked the ultrasound (n = 5), with one requesting additional ultrasound.

Participants in both groups provided positive feedback for treatment content and delivery (Supplementary Files 4 and 5, available at http://links.lww.com/PR9/A67). Suggestions to improve PSE content included simplifying concepts and checking in for understanding. Participants in both groups requested more follow-up (between end of treatment at 8 weeks and 6 month follow-up). Two participants (one/group) suggested group walking programs.

#### 3.2.2. Clinician-rated acceptability

Both clinicians rated that they thought participants in their respective groups found the interventions acceptable at 4 and 8 weeks, bar ratings for the 2 PSE participants who withdrew. Written feedback/interviews revealed that the PSE group had too much content (and focus on concepts/theory vs practical application), with insufficient time for discussion. In addition, the need to cover all PSE concepts for all participants was raised as problematic (not all topics apply to all people). It was highlighted that several participants were surprised that they would not receive “physiotherapy treatment” (ie, hands-on or specific exercise) and would only be receiving education (and walking program), with many expressing a desire for specific exercises at various times. In the Control group, the treating clinician reported that all treatment was received well and the amount of content appropriate. The Control group clinician also expressed challenges dealing with feelings of deceit, particularly when the participant attributed improvement to sham ultrasound.

### 3.3. Secondary outcomes

Barriers to participation related to the study time commitments (n = 1), no weekend appointments (n = 1), short time-interval of study (trip overseas, n = 1), undertaking other treatment (n = 2), not wanting an x-ray to participate (n = 2), and reason unknown (n = 2; 1 × nonattendance at baseline). The within-group changes for all clinical and activity outcomes are listed in Table 5 (see Supplementary File 6 for sensitivity analyses, available at http://links.lww.com/PR9/A67). Generally, these exploratory comparisons indicated that the PSE intervention increased pain knowledge, reduced unhelpful pain beliefs, and had positive effects on pain, function, and walking (particularly at 8 weeks).

**Table 5:** Clinical and physical activity outcome within group change scores and 95% confidence intervals.

	Pain science education			Control		
	Baseline—4 wk	Baseline—8 wk	Baseline—26 wk	Baseline—4 wk	Baseline—8 wk	Baseline—26 wk
Avg pain (rest) most painful knee	−1.8 (−3.1 to −0.6)*	0.4 (−2.2 to 1.4)	−0.9 (−2.7 to 0.9)	−1.7 (−3.9 to 0.4)	−2.2 (−4.4 to −0.2)*	−2.3 (−5.7 to 1.2)
Avg pain (walking) most painful knee	−1.8 (−3.3 to −0.2)*	−1.7 (−3.3 to −0.1)*	−0.9 (−2.9 to 1.2)	−1.9 (−3.2 to −0.5)*	−2.7 (−4.4 to −1.0)*	−2.8 (−5.5 to 0.0)
Avg pain (rest) least painful knee	−1.7 (−3.6 to 0.2)	−0.8 (−3.6 to 1.9)	−1.0 (−3.5 to 1.6)	−2.1 (−4.1 to −0.1)*	−2.1 (−5.7 to 1.5)	−2.9 (−26.4 to 20.6)†
Avg pain (walking) least painful knee	−1.0 (−4.3 to 2.2)	−2.4 (−4.9 to 0.1)	−1.0 (−3.4 to 1.5)	−0.8 (−3.9 to 2.3)	−1.0 (−6.9 to 4.8)	−3.9 (−55.2 to 47.4)†
WOMAC overall	−15.3 (−24.4 to −6.2)	−12.9 (−22.8 to −2.9)*	−11.0 (−22.7 to 0.7)	−8.4 (−18.3 to 1.6)	−11.2 (−23.8 to 1.4)	−5.1 (−19.4 to 9.1)
WOMAC pain	−3.9 (−6.7 to −1.1)*	−3.1 (−4.9 to −1.4)*	−2.0 (−4.3 to 0.3)	−2.4 (−5.0 to 0.3)	−3.1 (−6.3 to 0.2)	−2.7 (−6.7 to 1.2)
WOMAC function	−11.4 (−18.8 to −4.1)*	−9.8 (−18.7 to −0.9)*	−9.0 (−19.9 to 1.9)	−6.0 (−13.8 to 1.8)	−8.2 (−18.6 to 2.3)	−2.4 (−13.0 to 8.1)
PSFS activity 1	0.9 (−0.27 to 2.0)	1.7 (0.1 to 3.4)*	0.8 (−2.2 to 3.8)	0.9 (−1.0 to 2.8)	0.8 (−2.1 to 3.7)	0.6 (−2.0 to 3.3)
PSFS activity 2	0.9 (0.03 to 1.7)*	1.3 (0.4 to 2.2)*	1.2 (−1.2 to 3.6)	1.7 (−0.6 to 3.9)	1.1 (−1.5 to 3.7)	1.5 (0.1 to 2.9)
PSFS activity 3	0.9 (0.03 to 1.7)*	1.3 (0.3 to 2.3)*	0.6 (−2.8 to 3.9)	0.8 (−1.6 to 3.1)	1.3 (−2.4 to 4.9)	2.2 (−0.02 to 4.4)
PSEQ	12.8 (4.6 to 20.9)*	9.9 (4.3 to 15.5)*	8.7 (0.1 to 17.4)*	2.9 (−1.1 to 6.8)	4.1 (0.5 to 7.7)*	−1.4 (−8.3 to 5.6)
Brief FoM	0 (−3.5 to 3.5)	−1.5 (−4.9 to 1.9)	2.4 (−1.1 to 5.9)	−2.2 (−4.0 to −0.4)*	2.4 (−5.3 to 0.5)	−3.0 (−6.5 to 0.5)
PCS	2.9 (−3.9 to 9.8)	−1.1 (−7.4 to 5.3)	1.3 (−6.1 to 8.7)	−3.4 (−7.5 to 0.7)	−4.8 (−10.4 to 0.8)	−6.3 (−17.1 to 4.5)
PBQ—Organic	8.4 (1.6 to 15.2)*	9.0 (3.4 to 14.6)*	6.1 (0.9 to 11.4)*	2.5 (−0.5 to 5.6)	1.9 (−1.4 to 5.2)	4.4 (1.3 to 7.5)*
PBQ—Psych	−0.9 (−4.9 to 3.1)	0.4 (−2.8 to 3.6)	0.3 (−4.5 to 5.1)	−1.9 (−5.1 to 1.4)	−1.3 (−4.5 to 1.9)	−0.2 (−4.0 to 3.6)
rNPQ	2.8 (0.6 to 4.9)*	3.0 (1.2 to 4.8)*	3.0 (0.5 to 5.5)*	0.7 (−0.3 to 1.7)	0.9 (−0.1 to 1.9)	0.7 (−1.2 to 2.6)
Avg daily step count (steps/d)	N/A	1876 (551 to 3201)	614 (−626 to 1854)	N/A	86 (−1511 to 1682)	958 (−869 to 2785)
Avg daily sedentary time (min/d)	N/A	−24 (−148 to 99)	−25 (−143 to 93)	N/A	−81 (−150 to −11)*	−65 (−120 to −10)*
Avg daily light time (min/d)	N/A	54 (3 to 106)	443 (−27 to 114)	N/A	6 (−67 to 81)	43 (−7 to 92)
Avg daily mod time (min/d)	N/A	28 (−2 to 58)	16 (−9 to 41)	N/A	28 (2 to 54)*	23 (−5 to 50)

Pain science education, Baseline—4 weeks (n = 8, except pain for least painful knee, PCS, and PSFS: n = 7); 8 weeks (n = 8, except least painful knee and PSFS: n = 7); 26 weeks (n = 9, except pain for least painful knee, most painful knee walking, PSFS activity 1: n = 6; PSFS activity 1 and 2: n = 5). Control, Baseline—4 weeks (n = 10, except PSFS activity 1 and 2: n = 9; PSFS activity 3: n = 8; pain for least painful knee: n = 5); 8 weeks (n = 9, except PSFS activity 3: n = 7; pain for least painful knee: n = 3); 26 weeks (n = 7, pain for least painful knee, n = 2; PSFS activity 2, n = 6, PSFS activity 3, n = 5).

* Statistically significant within group change (confidence intervals do not include zero).

† Only n = 2.

Avg, average; Brief FoM, brief fear of movement scale; PBQ, pain beliefs questionnaire; PCS, pain catastrophizing scale; PSFS, patient-specific functional scale; PSEQ, pain self efficacy questionnaire; Psych, psychological rNPQ, revised neurophysiology of pain questionnaire; WOMAC, Western Ontario McMaster Universities OA index.

## 4. Discussion

This study aimed to evaluate the feasibility of an RCT investigating the addition of PSE (vs sham ultrasound) to a general OA/activity education and individualised walking program. Feasibility criteria related to recruitment, intervention adherence, and compliance with objective activity measures were met. Retention to longer follow-up was not considered adequate, with changes needed before undertaking a full trial. Treatment content and delivery mode were viewed positively in both groups, although both participants and the clinician in the PSE group highlighted that reduced content was needed. Generally, within-group changes support the ability of a walking program to increase activity in the short term, but not long term (26 weeks). Barriers to participation seem primarily related to features inherent to a feasibility study (eg, no weekend appointments), although unwillingness to undergo an x-ray (22% declined participation) is an important consideration for the full trial.

The significant interest to participate in this study by people with knee OA, as evidenced by high recruitment rates, supports progression to a large clinical trial. Indeed, although practicalities (funding/personnel) limited the number of participants/group that could be included, advertisements (newsletters and opportunistic television clip) resulted in a wait-list of 100. Also relevant to consider is whether people who would benefit most from a walking program (inactive) were recruited. Although participants' activity levels seem high (average baseline step count of >10,000), this is likely an artefact of the wrist-based accelerometry protocol—a recent study found that wrist-worn Actigraph accelerometers overestimated step count (vs hip-based accelerometers) by nearly a factor of two.^21^ Thus, while we recruited participants who would experience health benefits from increasing activity, further refinement of activity eligibility criteria (ie, using a maximal walking distance/duration exclusion) to recruit those most likely to benefit from the intervention is likely warranted.

Strategies to improve retention are needed. To reduce withdrawal from PSE treatment, 2 features seem important: (1) screening for past physiotherapy treatments received for back pain (the primary condition for which PSE is also provided^25,28,29^) and (2) updating the intervention description on the participant information sheet to minimise influences (and violations) of patient expectations of what physiotherapy involves (eg, “education and walking program” vs “physiotherapy treatment”). To reduce general loss to follow-up, ensuring sufficient time is taken to detail the study requirements and the importance of continued follow-up may be relevant. Some loss to follow-up was due to ongoing medical issues (ie, comorbidities), so planned flexibility in outcome assessment timing for a larger trial may be important to minimise missing data.

Objective assessment of physical activity through wrist-worn accelerometry was feasible. Compliance was high, no accelerometers were lost during mail-out, and there was only one accelerometer malfunction. Differences in group activity outcomes as a function of the analysis type, where step count favours the PSE group and activity count favours the Control group, highlight potential differences in walking program prescription, perhaps due to baseline activity differences (despite excluding those meeting moderate-vigorous activity guidelines). Refinement of the activity eligibility criteria as mentioned above and use of numerous treating clinicians per intervention (to avoid any clinician-specific group differences) seem most relevant. Furthermore, inclusion of additional in-person appointments in a future RCT, as requested by participants, seems important to promote longer-term walking increases.

Based on participant and clinician feedback, changes to PSE content are warranted. Specifically, the curriculum of PSE needs to be simplified and individualised to avoid a didactic educational session and to provide adequate time for discussion of complex concepts. Low PSE treatment credibility/acceptability ratings by participants suggests that more care needs to be taken to present education as an intervention itself, as is recommended in clinical guidelines.^23^ Inclusion of traditional physiotherapy interventions recommended by current OA guidelines, such as strengthening exercises,^23^ may assist in better matching patient expectations of physiotherapy and provide better generalisability. Revision of the intervention for a full trial (including PSE and activity content) would benefit from codesign with relevant end-users, most notably, people with symptomatic knee OA.

Participants had high credibility ratings for the Control intervention, confirming that sham ultrasound is a credible placebo. Different placebo interventions have different effects (some stronger than others).^18,19^ Consequently, ultrasound may not be an ideal sham to match the “talking/education” aspect of PSE, given that ultrasound is a passive, hands-on treatment that also matches participant's expectations of physiotherapy. Combined with the Control clinician's feedback (ie, feelings of deceit) and challenges implementing sham ultrasound in multiple clinics in a larger trial, it may be relevant to avoid its use. A more clinically relevant comparison would evaluate the benefit of adding intensive PSE to usual guideline-based care (education, walking program, and strengthening exercise). Such a comparison would provide direct evidence to support (or refute) implementation of the intervention into routine management of knee OA.

Strengths of this study include thoroughly exploring feasibility to ensure appropriate use of future research resources, using a high-quality design following CONSORT^14,40^ and TiDieR^17^ statements, and successful sham. Limitations are those inherent to feasibility studies: the results may not necessarily generalise to larger-scale trial results, particularly multisite trials. Furthermore, it is a small study, meaning that it is not powered for efficacy analyses. Caution in interpreting within-group data is also needed, particularly given baseline differences between groups (eg, WOMAC score), which can influence the impact of treatment on clinical outcome and on treatment credibility/acceptability ratings.

## 5. Conclusion

Feasibility criteria related to recruitment, intervention adherence, and compliance with objective physical activity assessment were met. Retention at long-term follow-up was not met (65% retained). Taken together, this feasibility study supports progression to a full trial by incorporating changes to increase participant retention, modifying PSE content and delivery, and better managing patient expectations.

## Disclosures

T.R. Stanton received travel and accommodation support from Eli Lilly Ltd for speaking engagements (2014; unrelated to the present topic). In the past 5 years, G.L. Moseley has received support from: ConnectHealth UK, Seqirus, Kaiser Permanente, Workers' Compensation Boards in Australia, Europe, and North America, AIA Australia, the International Olympic Committee, Port Adelaide Football Club, Melbourne Football Club, and Arsenal Football Club. Professional and scientific bodies have reimbursed him for travel costs related to presentation of research on pain at scientific conferences/symposia. He has received speaker fees for lectures on pain and rehabilitation. G.L. Moseley and D.S. Butler receives book royalties from NOIgroup publications, Dancing Giraffe Press & OPTP, including the 2 books used in the PSE group in this trial.

T.R. Stanton is supported by a National Health & Medical Research Council (NHMRC) Career Development Fellowship (ID1141735). G.L. Moseley is supported by an NHMRC Leadership Investigator Grant (ID1178444). K. Bennell is supported by a NHMRC Investigator Fellowship (ID1174431) and has received funding from the NHMRC and Medibank Private for research related to osteoarthritis. This work was supported by an Arthritis Australia & State/Territory Affiliate grant from Arthritis Australia (2018) and by an internal research grant from the Sansom Institute of Health Research, The University of South Australia. The funders had no role in study design, data collection and analyses, decision to publish, or preparation of the manuscript.

## Appendix A. Supplemental digital content

Supplemental digital content associated with this article can be found online at http://links.lww.com/PR9/A67.

## Supplementary Material

SUPPLEMENTARY MATERIAL
