# Supplementary material for: A pain science education and walking program to increase physical activity in people with symptomatic knee osteoarthritis: a feasibility study
Source: Pain Rep. 2020 Sep 24;5(5):e830. doi: 10.1097/PR9.0000000000000830 (PMC7808687; doi:10.1097/PR9.0000000000000830)
Supplement: SUPPLEMENTARY MATERIAL [file painreports-5-e830-s001.docx]

**Supplementary File 1: Enhanced education provided in Pain Science Education, including in-person sessions (Table 1) and at home activities between the in-person sessions (Table 2).**

| **THE ‘USUAL’ STORY OF KNEE OA**  **(Standard)**  *(From Arthritis Australia booklet)* | **Session** | **THE ‘MODERN’ STORY OF KNEE OA**  *The ‘usual’ story +*  ***current understanding of pain*** | **Session** |
| --- | --- | --- | --- |
| *What is osteoarthritis?*  OA is a condition that affects the whole joint including bone, cartilage, ligaments and muscles. | **1** | OA affects the whole joint – but it is a condition that also effects the *whole of* *you (*- it’s not just about ‘wear and tear’).  Wear is ok! | **2, 3 & 4** |
| OA tends to come on slowly. Joint pain or stiffness is usually worst with activity initially but can become more constant in later disease. | **1** | Progressive decline is not inevitable - even people with advanced OA can improve. | **2 & 4** |
| *What causes osteoarthritis?*  Risk factors for OA include being overweight, having a previous knee injury or a job involving lots of kneeling or squatting, and getting older. | **1** | Having risk factors for OA does not mean that you can’t improve with the right treatment. We all have ‘wrinkles on the inside’ (- aging is no excuse!) | **2 & 4** |
| Symptoms are variable but often affect your ability to do normal daily activities. | **1** | Bioplasticity means that being able to return to and gradually increase your daily activity is a reasonable expectation. | **3 & 4** |
| Your doctor may refer you for an x-ray of your knee (or another type of scan) to assist with the diagnosis of osteoarthritis. | **2** | The severity of changes shown on a knee x-ray do not have much relationship with how much pain you currently have or are likely to have in the future. Pain is complex and influenced by many things – not just what’s going on in your knee. | **2 & 3** |
| There is no cure for OA, but treatments can help to reduce symptoms and maintain function. | **2** | It may be beneficial to review current treatments based on new knowledge.  Learning about pain is an effective treatment – but learning and change takes time | **1 & 4** |
| If you are overweight, losing weight is key to managing osteoarthritis | **2** | Being overweight increases the load through your knee, but can also contribute to the progression of joint changes via the hormones/chemicals that circulate throughout your body if you are overweight. | **2, 3 & 4** |
| Doing regular physical activity can help to reduce your pain, strengthen your muscles, maintain your joint function and improve your sleep and overall health. | **3** | Regular physical activity has countless health benefits (at any age) and enhances bioplastic change in your *whole* system. This helps make your system *less* sensitive = able to do more with less pain.  There is very strong evidence that activity and exercise are safe and do not lead to further structural damage. | **3 & 4** |
| It is normal to feel some pain in your muscles when you start an exercise program or new activity. However, if pain feels unusual or severe, or lasts for more than 2 hours after you have stopped, it is probably best to avoid or change that activity. | **3 & 4** | Understanding what pain means can powerfully influence pain.  It is often not necessary to stop an activity if it is painful.  Your own brain can make powerful medications to reduce pain. | **All** |

**Table 1.**  Educational topics in the Usual care [standard education] control group and enhanced education provided in the Pain Science Education intervention group

| **MESSAGE** | | |
| --- | --- | --- |
| **Target Concept** (Linked to Objectives) | **Content**  (Syllabus) | **Delivery & Resources**  (Homework tasks in ***bolded italics***) |
| **Introduction to Pain Education (beginning of session 1):**   - Holistic confrontation: pain mechanisms model (educationally sound) – a ‘big picture’ overview (visual model/drawing). - Link to what is to follow…. talk about 4 visits… What will I do?/What will you do? (provide general overview, discuss goals and expectations) | | |
| **1. Learning about pain can help reduce pain and enhance physical activity** | - There is evidence that PSE is beneficial for in other conditions - and the evidence also suggests that it will work for people with knee OA - Knowledge itself can be analgesic - Knowledge can open the ‘drug cabinet in the brain’ - Pain education is relevant for *all* pain - Knowledge changes the way you think about movement - The right knowledge can allow you to move very differently | - EP p. 8-11 - EP evidence (EPS Ch. 4; other relevant studies) - Knowledge is analgesic (EPS Nugget 54, p.190) - ***Homework:*** - ***EPH 112-113: ‘Tool 1: Education and understanding’*** - ***EPH 5*** |
| **2. Pain is always real** | - You are not alone (e.g. 1 in 2 people >65yrs and 4m adults of working age have painful OA) - Emotional and physical pain are intertwined - There is no test for pain or love - You are the expert on your pain - Pain is a ‘gift’ and is necessary for survival | - Statistics for OA/OA Knee – including OA pain epidemiology - Congenital analgesia example. - Picture of a fMRI image of pain in the brain (orient participants to brain areas) - EPS Nugget 2 p.175 (Pain is a defender not an offender) |
| **3. There are danger detectors, not pain detectors** | - There are no pain endings, pain fibres, pain pathways or pain signals - The danger alarm system - Danger detectors: there are lots of them and they can change - Danger messages can be altered in many places (in the tissues, the spinal cord and in the brain) - Danger detectors can be active all the time – but we are not necessarily in pain all the time (give non-knee example – and then link to knee via discussion of low grade inflammation). - ‘Danger’ signals from your knee are represented in your brain (re-visit: the knee in the brain) | - EPH 7 (no hunger signals), 8-9 (info to fill in on their own, pain not relating to damage) - EP 28-39 - Draw the danger pathway and discuss input types (chemical, mechanical, temp) - EPS Nugget 19, p.178 (Lucky us – no pain endings); video clip for this one. - EPS Nugget 18, p.177 (Danger detectors – the great givers of life) |
| **Session 1 re-cap:**   - Knowledge is power – the more you understand the more helpful it will be. It will help future-proof you… - Your pain is mostly invisible but we know for sure that it’s real - We also know that impulses from your knee tell you about danger – but those messages on their own are not enough to make pain. - Let’s go back to the drawing… the key thing to understand is that humans are completely adaptable! There are so many places that your system can change (your knee can get healthier, with healthy movement and less stress the brain can change…and then what the brain produces can further change things in the knee…. - You are starting to understand these things so you are on your way…. | | |
| **Revision (beginning of session 2):** What did you take from last week? Could you describe what you learned to someone else?  Reflection on readings, re-cap on ‘danger’ detector/messages/pathways | | |
| **4. Radiographic changes usually do not relate to pain or to prognosis**  **&**  **What else is going on in the tissues and how does it relate to pain?** | - Identifiable changes on x-ray are *not* pain and many people without pain have these changes too - Amazing pain stories - Pain is an unreliable indicator of tissue damage - We don’t treat x-rays or scans - When *are* scans important? - ‘Phew, it’s only arthritis! - We will offer an alternative (more complete) explanation for your OA pain. (- promise!) | - EP p. 12-15 (Amazing pain stories) - Stages of OA and % without pain (stats/graph) - Positive scans in pain-free people (x-rays) - News story of the runner with ‘terrible’ knees - Scans are often important in the case of suspected # (following a fall) or suspicion of serious pathology (rare) - EPS Nugget 16 p.177 (We stop feeling it way before we stop healing it) - EPS Nugget 67 p. 194 (We grow like trees) - *If participants bring up MRI findings, have resources/explanation ready |
| **5. Pain is completely dependent on context** | - What is distributed brain activity? - Thoughts and beliefs are nerve impulses too (linked to distributed brain activity) - What is context? (The current situation you are in; your temporary state that is informed by everything going on *in and around* you: the things you hear, see, smell, taste and touch; the things you do and things you believe…). - Context, like your pain experience, is individual and unique - Usually you are not fully aware of context and its effect - Knowledge is context too - Short mirror neuron explanation/example/story. | - Complete the previous danger pathway drawing to include the brain - EPH 11 (why do I hurt) - EP 18-21 - EPS Nugget 46 p.188 (Plumbers and poos, electricians and zaps) - EP 38-41 (distributed processing) - Images which depict complex, interconnected dynamic networks (e.g., airline flight paths, the brain as an orchestra) - EPS Nugget 5 p. 174 (Grandma is distributed in the brain) - Mirror neuron activity: imagine a past experience of being active; moving easily and pain free….or reflect on how your knee might hurt if you even think about moving in a certain way. |
| **6. Pain depends on the balance of danger and safety - PART 1** | - Modern pain definitions - Introduce DIMs, SIMs and DIM/SIM balance - Introduce DIM/SIM part of the Protectometer - The ‘on alert’ zone (between 0 and 1 on the protectometer – have I got a headache or not?) - DIMs and SIMs hide in hard to find places - Danger and safety ‘patterns of brain activity’ - Link to patterns of brain activity related to danger signals from your knee (unity of systems) | - EPH 13-23 (DIMs/SIMs & Protectometer) - Lorimer Moseley’s TED talk: snake story - EPS Nugget 50 p.189 Bilby in the bath – - Work through DIMs and SIMs relevant to walking - Exercise – danger/safety wheel (EPH p. 15); links to individualised context - Danger/safety specific to physical activity - ***Homework: Personal Protectometer at home - consider DIMs and SIMs more broadly*** |
| **Introduction to bioplasticity (Target concept 9.)** | - Reflect on the power of healing |  |
| **Session 2 re-cap:**   - Scans are only one part of the story. What’s going on in your knee may be responsible for danger signals but these can be modified by everything else that is going on in your system and your brain. X-rays usually don’t relate to the pain that you feel or how much pain you will have in your future – because the changes in your knee are only a tiny part of you and are a normal adaptation to life. - We are bioplastic: pain can be modified by anything – even changing the way you think about what’s going on in your knee can change the pain that you feel. But the pain you experience with your osteoarthritis is about so much more than your knee… | | |
| **Revision (beginning of session 3)**: Work through DIMs/SIMs identified in homework task. | | |
| **6. Pain depends on the balance of danger and safety – PART 2** | - Explore DIMs and SIMs at play more broadly - DIMs and SIMs often hide in hard to find places - SIMS open up the drug cabinet in the brain, DIMS close the door. - There is a neuroscience rationale for increasing SIMs to reduce pain - Return to the OVERVIEW DIAGRAM and add anything new that comes up from review of DIMs and SIMs - Discuss changes in/at the synapse | - Consider context in walking program: boosting SIMs - Identify super-DIMs and break them down? - Some DIMs can’t be changed, but acknowledging them and understanding that they have impact can change them or alter their impact - Identify super-SIMS - Give them a DIM/SIM challenge(s) |
| **7(a). The pain system can become overprotective** | - The pain protective system can become “turned up and edgy” - Overprotection is understandable; it makes you super safe (the brain is actually trying to help!) - Increased protective, safety buffer. - The “orchestra” repeats the pain and only plays the pain tune - Flare-ups are inevitable, common and understandable; understanding about them helps them resolve quicker - You are able to ‘wind down’ this overprotection (movement and knowledge are highly important) | - EP 70-79, 82-83 (The sensitised central alarm system) - Refer to DIAGRAM and add to it to show enhanced sensitivity in the dorsal horn and the brain - ***Homework:*** - ***Kangaroo tracks in the brain*** - ***Twin peaks model EP p. 119*** |
| **7(b). Pain is only one of many protective outputs that can also become over-protective** | - Introduction to other protective systems including endocrine, immune, motor, cognitive, respiratory, sympathetic. - The increased sensitivity of the protective system also involves body systems apart from pain (immune, endocrine, etc.) - Inflammation, movement and stiffness are protective outputs too, e.g. knee giving way - Protective systems have an adaptive purpose – but they can become unhelpful when they are overprotective - Your thoughts and emotions are protective outputs too - ? Focus on endocrine system as an example | - EPH p. 24: blue brain; - EPH p. 27: pink brain - EPS Nugget 35 p. 184 (Stress and swelling) - EPS Nugget 38 p. 185 (rest and digest) - EPS Nugget 42 p. 186 (fingernails grow faster on holidays) - EPS Novella 10 p. 213 (Protectometer for stress, fatigue and anxiety) – if relevant - ***Homework: Turned up, edgy systems’ exercise (EPH 27)*** |
| **Re-cap session 3**:   - When the DIM SIM balance isn’t ideal, body systems can become overprotective. - The pain system can become overprotective at many levels of the system – but you are capable of ‘winding down’ the protective system. You have your own personal drug cabinet in your brain and the DIM/SIM balance determines whether the door is open or closed. There are many ways to start to change this balance…. - There is a good and a bad side to bioplasticity – new pathways can be formed in the brain | | |
| **Revision (beginning of session 4):** Check in – how are they going? Seek self-explanation. Revise list of target concepts and what they mean for the individual.  Further discuss the importance of context in making progress with activity/exercise goals | | |
| **9. We are bioplastic throughout our lifespan**  **INTRODUCED IN PREVIOUS SESSIONS: Emphasis is now on activity-focussed bioplasticity** | - What is bioplasticity? - Bioplasticity occurs in the brain’s representation of the knee and in the knee structure itself (link to ‘wear’) - Bioplasticity occurs in all body systems, throughout the lifespan - There is a dark and a bright side to bioplasticity - Why understanding bioplasticity increases hope - The number of danger detectors (and their sensitivity) can change; DRH can change (# of receptor and sensitivity); brain can change (structural and functional changes; DIMs/SIMs) - Virtual body and homunculus – the knee in the brain (link to changes in motor function and activity). | - EPH 30-32 - EPS Novella 14 p.218 (Your ever changing brain) - Example of an older person taken up sport (or learning new skills) - Dealing with DIMs |
| **10. Active treatment strategies promote recovery** | - Evidence-based treatment options for OA. - Contrasting active and passive treatments - Move smart (pacing and not overdoing it, moving is learning) - Goal setting - Use your own drug cabinet (endogenous versus exogenous medication) - Have optimal linguistic expression in relation to OA and activity (e.g. avoid “wear and tear”) - The place of pills - Exercise and activity enhance bioplastic change and have an important impact on your safety buffer - Flare-ups are common (and may enhance bioplastic change). Discuss strategies for what to do in the case of a flare up. | - Example of what active and passive means - EP 80-81, 94-107 - EPH 34-48 - ***Homework: YouTube: ‘Brainman stops his opioids’ and ‘Drug cabinet in the brain’*** - EPS Novella 9 p. 212 (Movement, the SIM-fest!) - Re-visit twin peaks model - ***Homework: EP 116-119: pacing and graded exposure*** |
| **SUMMARY:**  **The modern story of osteoarthritis** | 1. OA affects the whole joint – but it is a condition that also effects the whole of you (- it’s not just about ‘wear and tear’). Some wear is ok! 2. Progressive decline is not inevitable - even people with advanced OA can improve. 3. Having risk factors for OA does not mean that you can’t improve with the right treatment. We all have ‘wrinkles on the inside’ (- aging is no excuse!) 4. Bioplasticity means that being able to return to and gradually increase your daily activity is a reasonable expectation. 5. The severity of changes shown on a knee X-Ray do not have much relationship with how much pain you currently have or are likely to have in the future. Pain is complex and influenced by LOTS of things – not just what’s going on in your knee. 6. It may be beneficial to review current treatments based on new knowledge. Learning about pain is an effective treatment – but learning and change takes time…. 7. Being overweight increases the load through your knee, but can also contribute to the progression of joint changes and via the hormones/chemicals that circulate throughout your body if you are overweight. These also influence your sensitivity. 8. Regular physical activity has countless health benefits (at any age) and enhances bioplastic change in your whole system. There is very strong evidence that activity and exercise is safe and does not lead to further structural damage. 9. Understanding what pain means can powerfully influence pain. It is often not necessary to stop an activity if it is painful. Your own brain has powerful mechanisms of reducing pain. | |

**Table 2.** In-depth session specific Pain Science Education intervention, including homework. OA, Osteoarthritis; EP, Explain Pain; EPS, Explain Pain Supercharged; EPH, Explain Pain Handbook.
