# Supplementary material for: A pain science education and walking program to increase physical activity in people with symptomatic knee osteoarthritis: a feasibility study
Source: Pain Rep. 2020 Sep 24;5(5):e830. doi: 10.1097/PR9.0000000000000830 (PMC7808687; doi:10.1097/PR9.0000000000000830)
Supplement: SUPPLEMENTARY MATERIAL [file painreports-5-e830-s002.docx]

**Supplementary File 2: In-person treatment session by session breakdown for each group.**

| **Treatment component** | **Interventions - Graded walking program combined with:** | | **Timing Considerations** |
| --- | --- | --- | --- |
|  | ***Standard Education + Sham US (Control)*** | ***Enhanced education (PSE)*** |  |
| **Session 1** | | | To more closely match treatment duration and therapist time between groups, the Control group will have:  1. Increased time taken for the Standard physiotherapy assessment (go slow!)  2. Inactive ultrasound application |
| Baseline assessment | Standard physical examination  Standard subjective examination | Standard physical examination  Enhanced subjective examination (‘standard’ + identification of participants’ education targets for PSE). |  |
| Education | General education  Provide inactive ultrasound to 4 locations on the most painful knee (~5 mins each).  Introduce participants to the AA handbook (to take home) | Enhanced education  Introduce participants to ‘Explain Pain Handbook’ and the ‘The Protectometer’ (to take home) |  |
| Graded walking program | Instructions for following week re: Establish baseline walking tolerance that does not result in sustained, increased pain following walking (for next session) | |  |
| **Session 2** | | | For Sessions 2-4:  To more closely match treatment duration and therapist time between groups, the Control group will have:  1. A longer re-assessment at the beginning of the session  2. Inactive ultrasound application |
| Assessment | Standard subjective and physical re-assessment | Brief subjective evaluation and re-cap of session 1 |  |
| Education | General education  Discussion of x-ray findings (read interpretation section)  Provide inactive ultrasound to 4 locations on the most painful knee (~5 mins each). | Enhanced education  Discussion of x-ray findings (focus on normal age-related changes; positive reframing of structural findings) |  |
| Graded walking program, goal-setting | Use baseline walking tolerance to calculate a ‘start’ walking level and set a walking program for the week ahead.  Set activity goals (short and long term) | |  |
| **Session 3** | | |  |
| Assessment | Standard subjective and physical re-assessment | Brief subjective evaluation and re-cap of sessions 1&2 |  |
| Education | General education  Provide inactive ultrasound to 4 locations on the most painful knee (~5 mins each). | Enhanced education |  |
| Graded walking program | Check in. Increase activity by 10% during the next week.  Discuss general principles of activity pacing.  Provide the Practical considerations of activity handout | |  |
|  | N/A | Discuss and consider context when planning walking/activity this week |  |
| **Session 4** | | |  |
| Assessment | Standard subjective and physical re-assessment | Brief subjective evaluation and re-cap of sessions 1-3 |  |
| Education | General education  Provide inactive ultrasound to 4 locations on the most painful knee (~5 mins each). | Enhanced education |  |
| Graded walking program, goal-setting | Check in. Increase activity by 10% during the next week.  Set-up walking and general activity plan over the next 4 weeks. | |  |
|  | Discuss flare-ups and how to reduce activity. | Review flare-ups – what they mean (pain science) and the activity plan. |  |

US, Ultrasound; PSE, Pain Science Education; AA, Arthritis Australia.
