# Supplementary material for: A pain science education and walking program to increase physical activity in people with symptomatic knee osteoarthritis: a feasibility study
Source: Pain Rep. 2020 Sep 24;5(5):e830. doi: 10.1097/PR9.0000000000000830 (PMC7808687; doi:10.1097/PR9.0000000000000830)
Supplement: SUPPLEMENTARY MATERIAL [file painreports-5-e830-s003.docx]

**Supplementary File 3: Questionnaires to gather participant and clinician perceptions on intervention acceptability**

**Participant Experience Questionnaire**

(Credibility – 1, 2, 3; Acceptability – 4, 5, 6; Perceived usefulness – 7, 8, 9, 10)

**Consider the following statements and place a tick in the box that best describes your response:**

|  | **Strongly disagree** | **Disagree** | **Unsure** | **Agree** | **Strongly agree** |
| --- | --- | --- | --- | --- | --- |
| 1. I would recommend this treatment to other people with knee osteoarthritis |  |  |  |  |  |
| 1. I had confidence in the expertise of the Physiotherapist who treated me |  |  |  |  |  |
| 1. It was easy to believe what the Physiotherapist told me |  |  |  |  |  |
| 1. I enjoyed attending the treatment sessions |  |  |  |  |  |
| 1. The treatment sessions were relevant to me |  |  |  |  |  |
| 1. It was worthwhile attending the treatment sessions |  |  |  |  |  |
| 1. The treatment sessions have increased my knowledge and understanding |  |  |  |  |  |
| 1. As a result of the treatment sessions I am likely to increase my activity level in the **short** term (the next 3-6 months) |  |  |  |  |  |
| 1. As a result of the treatment sessions I am likely to increase my activity level in the **long** term (beyond 3-6 months) |  |  |  |  |  |
| 1. The treatment sessions have changed the way I think about my knee pain |  |  |  |  |  |

**Participant short answer questions**

**Please provide a short answer to the following questions:**

1. What did you like most about the treatment that you received?

________________________________________________________________________________________________________________________________________________________________________________________________________________________________________

2. What did you like least about the treatment that you received?

_____________________________________________________________________________________________________________________________________________________________________________________________________________________________________

3. Do you have any suggestions for how the CONTENT of the treatment sessions could be improved?

____________________________________________________________________________________________________________________________________________________________________________________________________________________________________

4. Do you have any suggestions for how the FORMAT of the treatment sessions could be improved? (e.g. number of sessions, duration of sessions)

____________________________________________________________________________________________________________________________________________________________________________________________________________________________________

**Clinician Short Answer Questions**

**After each participant:**

1. Do you think the participant considered the intervention to be an acceptable treatment?

Yes / No (circle). If ‘No’: please describe any aspects that were not well accepted.

_____________________________________________________________________________________________________________________________________________________________________________________________________________________________________

**At the end of the study:**

**Please provide a short answer to the following questions:**

1. Briefly describe your experience as the therapist delivering the Pain Science Education/Control intervention in this study:

________________________________________________________________________________________________________________________________________________________________________________________________________________________________________

2. Do you have any suggestions for how the CONTENT of the treatment sessions could be improved?

____________________________________________________________________________________________________________________________________________________________________________________________________________________________________

3. Do you have any suggestions for how the FORMAT of the treatment sessions could be improved? (e.g. number of sessions, duration of sessions)

____________________________________________________________________________________________________________________________________________________________________________________________________________________________________

4. Do you have any other suggestions?

____________________________________________________________________________________________________________________________________________________________________________________________________________________________________
