# Supplementary material for: A pain science education and walking program to increase physical activity in people with symptomatic knee osteoarthritis: a feasibility study
Source: Pain Rep. 2020 Sep 24;5(5):e830. doi: 10.1097/PR9.0000000000000830 (PMC7808687; doi:10.1097/PR9.0000000000000830)
Supplement: SUPPLEMENTARY MATERIAL [file painreports-5-e830-s004.docx]

**Supplementary File 4: Content analysis of the participant short questionnaires at 4 weeks, 8 weeks and 6 months.**

| **FEEDBACK FORMS** | **PAIN SCIENCE EDUCATION** | | | **CONTROL** | | |
| --- | --- | --- | --- | --- | --- | --- |
|  | **Week 4** | **Week 8** | **6 month** | **Week 4** | **Week 8** | **6 month** |
| **Likes** | 3 x friendly staff  3 x increasing knowledge of pain  2 x personal interaction  2 x teaching of content  1 x Non-invasive  1 x set goals  1 x importance of keeping active  1 x confidence | 2 x at home  2 x Focused goals  1 x motivation  1 x DIM/SIM daily  1 x awareness  1 x Flexible schedule  1 x importance of home tasks | 4x Knowledge of pain  3 x benefits  2 x not considered ‘treatment’  1 x importance of keeping active  1 x teaching style | 5 x knowledge- exercise is safe  4 x Confidence  3 x professional staff  2 x Friendly  2 x ultrasound  1 x gait improvements  1 x benefits (hope) | 3 x goals specific & manageable  3 x consistent walking  2 x Encouragement  2 x Confidence  1 x at home (easier)  1 x make time for activity | 2 x Confidence  2 x benefits (hopeful)  1 x thankful for opportunity  1 x decrease pain |
| **Dislikes** | 7 x nothing  1 x takes time to see change | 3 x None  2 x time (similar questions each week)  1 x heavy concepts (struggled to understand)  1 x Missed call (disappointed) | 4 x None  1 x travel to uni  1 x time required for follow up | 4 x Nothing  3 x no answer  2 x repetition  1 x squats | 3 x nothing  2 x no answer  1 x paperwork  1 x repetition  1 x missed face to face contact  1 x ongoing time commitment | 1 x minimal improvement  1 x getting to sessions  1 x pushing through pain  1 x time required  1 x lost confidence  1 x nothing |
| **Content** | 3 x No changes needed  2 x No response  1 x too early to say (want whole program 1^st^)  1 x exercises with physio  1 x use a powerpoint or display | 5 x no changes  1 x use medical drawings  1 x manageable currently  1 x simplify (wording) | 3 x No changes needed  1 x Explain plain book good  1 x increased pain medication/surgery discussion  1 x simplify book | 6 x No changes needed  1 x repetition  1 x testimonies of similar patients | 3 x no changes needed  2 x No response  1 x sufficient currently  1 x specify self motivation needed for at home tasks  1 x more research summaries (study links) | 5 x no changes needed  1 x increased ultrasound  1 x more phone calls  1 x research summaries |
| **Improvement** | 2 x reasonable currently  1 x Probe questions- check patient understands  1 x structured appt times (limit to length)  1 x condense sessions | 1 x more calls  1 x folder/booklet to hold all worksheets  1 x wish it continued  1 x general discussion of weather impacting OA | 3 x no changes  1 x include pain log sheet to track improvements  1 x group session instead of individual  1 x more follow up | 5 x no changes needed  3 x no response  1 x walking group rather than individual  1 x condense content  1 x consistent time of appointments | 4 x no changes needed  1 x No response  1 x space appointments fortnightly  1 x reformat diary (more user friendly)  1 x consider impact of co-morbidities on participation | 5 x No changes needed  1 x increased contact in follow up period |
