# Supplementary material for: A pain science education and walking program to increase physical activity in people with symptomatic knee osteoarthritis: a feasibility study
Source: Pain Rep. 2020 Sep 24;5(5):e830. doi: 10.1097/PR9.0000000000000830 (PMC7808687; doi:10.1097/PR9.0000000000000830)
Supplement: SUPPLEMENTARY MATERIAL [file painreports-5-e830-s005.docx]

**Supplementary File 5: Content analysis of participant verbal interviews at 4 and 8 weeks.**

| **PHONE CALLS** | **PAIN SCIENCE EDUCATION** | | **CONTROL** | |
| --- | --- | --- | --- | --- |
|  | **Week 4** | **Week 8** | **Week 4** | **Week 8** |
| **General comments** | 6 x learning new information  5 x informative  5 x friendly/professional staff  4 x unexpected or non-traditional approach  3 x books excellent | 5 x happy  4 x homework  4 x more movement  2 x learning  2 x opportunity (thankful)  2 x walking  1 x bad pain  1 x okay  1 x weather effects  1 x sore at times | 7 x physio  6 x excellent experience  6 x increased confidence  5 x increased awareness  4 x ultrasound  3 x mindset shift  3 x professional  1 x unexpected  1 x frightened to move | 5 x positive  4 x pain limiting movement  4 x motivation  4 x physio  3 x good  3 x overdid it (+ new injury)  2 x mindset  2 x easy to follow  2 x informative |
| **Likes** | 5 x physio staff  3 x education component  2 x well-spaced content (small bites info)  2 x got me out of house  1 x Explain Pain concept (Pain Science Education)  1 x forced action  1 x increased motivation | 4 x at home flexibility (scheduling)  4 x self-motivating  3 x all good  1 x phone calls to clarify | 6 x increasing exercise (see improvement)  4 x no jargon (in books)  3 x all of it  3 x ultrasound  3 x having opportunity  1 x no use-by-date for knee | 6 x easy to follow  3 x reinforcing  3 x accountability  2 x checking workbook  1 x on-time |
| **Dislikes** | 5 x nothing  1 x building temperature (unclear but likely too cold)  1 x detailed information | 3 x none  2 x time to complete all tasks  1 x books wordy on own  1 x easier with physio for Qs | 7 x nothing  2 x repetition  1 x missing goals that had been set | 4 x repetitive  4 x miss ultrasound  2 x miss face to face  1 x paperwork |
| **Content** | 7 x very happy with current set up  1 x used to doing homework so no problem  1 x understand purpose now  1 x consider using a screen/computer display  1 x straightforward  1 x clearly presented | 4 x happy (no changes)  4 x straightforward  1 x clarify meaning ‘pacing- other activities’ when goal setting by self  1 x full on (level/amt of reading)  1 x needed self-discipline to complete | 7 x positive messaging is good  7x liked positive messaging  6 x no changes needed  5 x ultrasound good  2 x liked face to face | 4 x workbook good/clear  1 x exercises with physio (traditional model)  2 x easy to forget |
| **Improvement** | 2 x no changes  2 x face to face works to discuss Qs as come up  1 x shorter time period  1 x tell medical doctors about this  1 x exercises to do with physio (as supplement) | 8 x no changes | 6 x no changes needed  2 x parking  2 x more ultrasound  1 x repetition | 7 x no changes needed  1 x group activity  1 x consider mental health impacts of missing goals |
| **Travel/practicalities** | 6 x No issues  1 x sick-rescheduled no probs  1 x very professionally handled  1 x appointment rescheduled (preferred time not available)  1 x parking | 8 x no problems  1 x missed call- rescheduled easily | 4 x sessions too long  3 x used public transport  2 x credible because UniSA  1 x weekend call (good flexibility) | 5 x no problems  2 x phone good  1 x on-time  1 x flexibility of scheduling |
