# Supplementary material for: A pain science education and walking program to increase physical activity in people with symptomatic knee osteoarthritis: a feasibility study
Source: Pain Rep. 2020 Sep 24;5(5):e830. doi: 10.1097/PR9.0000000000000830 (PMC7808687; doi:10.1097/PR9.0000000000000830)
Supplement: SUPPLEMENTARY MATERIAL [file painreports-5-e830-s006.docx]

**Supplementary File 6: Sensitivity analysis for clinical and physical activity outcomes (baseline data carried forward)**

|  | **Pain Science Education** | | | **Control** | | |
| --- | --- | --- | --- | --- | --- | --- |
|  | Baseline - 4 wks | Baseline – 8 wks | Baseline – 26 wks | Baseline – 4 wks | Baseline – 8 wks | Baseline – 26wks |
| Avg pain (rest) most painful knee | **-1.5 (-2.6 to -0.4)*** | -0.3 (-1.7 to 1.1) | -0.6 (-1.8 to 0.6) | -1.7 (-3.9 to 0.4) | **-1.9 (-4.0 to -0.0)*** | -1.6 (-3.9 to 0.7) |
| Avg pain (walking) most painful knee | **-1.4 (-2.7 to -0.1)*** | **-1.4 (-2.7 to -0.1)*** | -0.5 (-1.6 to 0.6) | **-1.9 (-3.2 to -0.5)*** | **-2.4 (-4.0 to -0.8)*** | -1.9 (-3.9 to 0.1) |
| Avg pain (rest) least painful knee | -1.5 (-3.1 to 0.1) | -0.7 (-3.0 to 1.6) | -0.8 (-3.0 to 1.3) | **-2.1 (-4.1 to -0.1)*** | -1.3 (-3.2 to 0.7) | -1.2 (-3.7 to 1.4) |
| Avg pain (walking) least painful knee | -0.9 (-3.6 to 1.8) | -2.1 (-4.3 to 0.1) | -0.7 (-2.4 to 0.9) | -0.8 (-3.9 to 2.3) | -0.6 (-2.8 to 1.6) | -1.6 (-6.0 to 2.9) |
| WOMAC overall | **-12.3 (-20.5 to -4.0)*** | **-10.3 (-18.7 to -1.9)*** | **-**7.7 (-16.0 to 0.6) | -8.4 (-18.3 to 1.6) | -10.1 (-21.4 to 1.2) | **-**3.6 (-12.8 to 5.6) |
| WOMAC Pain | **-3.1 (-5.5 to -0.7)*** | **-2.5 (-4.1 to -0.9)*** | -1.4 (-3.0 to 0.2) | -2.4 (-5.0 to 0.3) | -2.8 (-5.7 to 0.2) | -1.9 (-4.6 to 0.8) |
| WOMAC Function | **-9.2 (-15.7 to -2.6)*** | **-7.8 (-15.1 to -0.5)*** | -6.3 (-13.8 to 1.2) | -6.0 (-13.8 to 1.8) | -7.4 (-16.7 to 2.0) | -1.7 (-8.4 to 5.0) |
| PSFS Activity 1 | 0.6 (-0.2 to 1.4) | 1.2 (-0.0 to 2.4) | 0.5 (-1.1 to 2.1) | 0.8 (-0.8 to 2.4) | 0.7 (-1.8 to 3.2) | 0.5 (-1.2 to 2.1) |
| PSFS Activity 2 | 0.6 (-0.0 to 1.2) | **0.9 (0.2 to 1.6)*** | 0.6 (-0.4 to 1.6) | 1.5 (-0.5 to 3.5) | 1.0 (-1.3 to 3.3) | 0.9 (-0.0 to 1.8) |
| PSFS Activity 3 | 0.6 (-0.0 to 1.2) | **0.9 (0.1 to 1.7)*** | 0.3 (-1.0 to 1.6) | 0.8 (-1.6 to 3.1) | 1.1 (-2.0 to 4.2) | 1.4 (-0.1 to 2.9) |
| PSEQ | **10.2 (2.9 to 17.5)*** | **7.9 (2.7 to 13.1)*** | 6.1 (-0.1 to 12.3) | 2.9 (-1.1 to 6.8) | **3.7 (0.4 to 7.0)*** | -1.0 (-5.4 to 3.5) |
| Brief FoM | 0.0 (-2.7 to 2.7) | -1.2 (-3.8 to 1.4) | 1.7 (-0.7 to 4.1) | **-2.2 (-4.0 to -0.4)*** | -2.2 (-4.8 to 0.4) | -2.1 (-4.6 to 0.4) |
| PCS | 2.1 (-2.4 to 6.5) | -0.9 (-5.6 to 3.9) | 0.9 (-3.8 to 5.6) | -3.4 (-7.5 to 0.7) | -4.3 (-9.3 to 0.7) | -4.4 (-11.5 to 2.7) |
| PBQ – Organic | **6.7 (1.0 to 12.4)*** | **7.2 (2.2 to 12.2)*** | **4.3 (0.4 to 8.2)*** | 2.6 (-0.5 to 5.6) | 1.7 (-1.3 to 4.7) | **3.1 (0.6 to 5.6)*** |
| PBQ – Psych | -0.7 (-3.7 to 2.3) | 0.3 (-2.1 to 2.7) | 0.2 (-2.9 to 3.3) | -1.9 (-5.1 to 1.4) | -1.2 (-4.0 to 1.6) | -0.2 (-2.5 to 2.2) |
| rNPQ | **2.2 (0.4 to 4.0)*** | **2.4 (0.7 to 4.1)*** | **2.1 (0.2 to 4.0)*** | 0.7 (-0.3 to 1.7) | 0.8 (-0.1 to 1.7) | 0.5 (-0.7 to 1.7) |
| Avg daily step count (steps/day) | N/A | **1313 (254 to 2372)*** | 368 (-301 to 1038) | N/A | 77 (-1323 to 1478) | 766 (-642 to 2175) |
| Avg daily sedentary time (mins/day) | N/A | **1313 (254 to 2372)*** | 368 (-301 to 1038) | N/A | 77 (-1323 to 1478) | 766 (-642 to 2175) |
| Avg daily light time (mins/day) | N/A | -17 (-95 to 61) | **-73 (-136 to -9)*** | N/A | -15 (-76 to 46) | **-52 (-98 to -6)*** |
| Avg daily mod time (mins/day) | N/A | **38 (0.3 to 76)*** | 6 (-59 to 71) | N/A | 26 (-14 to 65) | 33 (-6 to 73) |

**Table 1.** Baseline data carried forward for clinical and physical activity outcome within group change scores and 95% confidence intervals. Bolded entries represent significant within group change. * Statistically significant within group change (confidence intervals do not include zero). Pain Science Education, Baseline – 4 weeks (n=10, except least painful knee n=8); 8 weeks (n=10, except least painful knee n=8); 26 weeks (n=10, except least painful knee n=8). Control, Baseline – 4 weeks (n=10, except least painful knee n=5; PSFS Activity 3 n=8); 8 weeks (n=10, except least painful knee n=5; PSFS Activity 3 n=8); 26 weeks (n=10, except least painful knee n=5; PSFS Activity 3 n=8). WOMAC, Western Ontario McMaster Universities OA Index; PSFS, Patient-Specific Functional Scale; PSEQ, Pain Self Efficacy Questionnaire; Brief FoM, Brief Fear of Movement scale; PCS, Pain Catastrophizing Scale; PBQ, Pain Beliefs Questionnaire; rNPQ, Revised Neurophysiology of Pain Questionnaire.
